# TGF-β Promotes the Proliferation of Microglia In Vitro

**DOI:** 10.3390/brainsci10010020

**Published:** 2019-12-30

**Authors:** Costansia Bureta, Takao Setoguchi, Yoshinobu Saitoh, Hiroyuki Tominaga, Shingo Maeda, Satoshi Nagano, Setsuro Komiya, Takuya Yamamoto, Noboru Taniguchi

**Affiliations:** 1 Department of Orthopaedic Surgery, Graduate School of Medical and Dental Sciences, Kagoshima University, 8-35-1 Sakuragaoka, Kagoshima 890-8520, Japan; 2 Department of Orthopaedic Surgery, Japanese Red Cross Kagoshima Hospital, Kagoshima 891-0133, Japan; 3 Department of Medical Joint Materials, Graduate School of Medical and Dental Sciences, Kagoshima University, Kagoshima 890-8520, Japan

**Keywords:** transforming growth factor beta (TGF-β), galunisertib, microglia, central nervous system, proliferation

## Abstract

The activation and proliferation of microglia is characteristic of the early stages of brain pathologies. In this study, we aimed to identify a factor that promotes microglial activation and proliferation and examined the in vitro effects on these processes. We cultured microglial cell lines, EOC 2 and SIM-A9, with various growth factors and evaluated cell proliferation, death, and viability. The results showed that only transforming growth factor beta (TGF-β) caused an increase in the in vitro proliferation of both microglial cell lines. It has been reported that colony-stimulating factor 1 promotes the proliferation of microglia, while TGF-β promotes both proliferation and inhibition of cell death of microglia. However, upon comparing the most effective doses of both (assessed from the proliferation assay), we identified no statistically significant difference between the two factors in terms of cell death; thus, both have a proliferative effect on microglial cells. In addition, a TGF-β receptor 1 inhibitor, galunisertib, caused marked inhibition of proliferation in a dose-dependent manner, indicating that inhibition of TGF-β signalling reduces the proliferation of microglia. Therefore, galunisertib may represent a promising therapeutic agent for the treatment of neurodegenerative diseases via inhibition of nerve injury-induced microglial proliferation, which may result in reduced inflammatory and neuropathic and cancer pain.

## 1. Introduction

Microglia represent 5%–20% of the total glial cells in the central nervous system (CNS). They are immune-resident macrophages, which constantly patrol the CNS for plaques, damaged neurons, and infectious agents, reacting rapidly upon activation to minimize ensuing inflammation and damage [1,2,3].

Microglial proliferation and activation is observed following brain injury and in chronic inflammatory diseases such as Alzheimer’s disease, multiple sclerosis, and acquired immunodeficiency syndrome [2,3]. In these situations, resident ramified microglia transform into “activated microglia” [1]. Proliferating and activated microglia produce various chemical mediators including proinflammatory cytokines and chemokines with immunological actions, which act on neurons to alter their functions [4,5,6]. This alteration of neuronal function is key in the development of neuroinflammation and neuropathic pain [1,3].

Transforming growth factor beta (TGF-β) is a multifunctional cytokine [7]; members of the TGF-β family modulate the survival, activation, and proliferation of neural cells [8,9]. Galunisertib (LY2157299) is a selective adenosine triphosphate-mimetic inhibitor of TGF-β receptor I (TGFβRI), which is currently under clinical investigation for use in the treatment of myelodysplastic syndromes, glioblastoma, pancreatic ductal adenocarcinoma, metastatic pancreatic cancer, hepatocellular carcinoma, rectal adenocarcinoma, prostate cancer, and other solid tumours [10].

It is not clear what kind of signalling promotes microglial activation and proliferation; hence, we aimed to identify a signalling pathway that promotes microglial activation and proliferation in vitro. We found that TGF-β promotes the proliferation of microglial cells and that galunisertib attenuates this effect.

## 2. Materials and Methods

### 2.1. Cell Culture

The EOC 2 (ATCC^®^(American Type Culture Collection) CRL-2467™) and SIM-A9 (ATCC^®^ CRL-3265™) cell lines were purchased from ATCC (Manassas, VA, USA). We cultured EOC 2 cells in a complete growth medium, Dulbecco’s modified Eagle’s medium (DMEM), supplemented with 1.5 g/L sodium bicarbonate, 4.5 g/L glucose, 10% fetal bovine serum (FBS; Gibco, Thermo Fisher Scientific, Waltham, MA, USA), and 20% conditioned medium (produced from LADMAC cells (CRL-2420)). The SIM-A9 cells were cultured in DMEM supplemented with 10% FBS, 100 U/mL penicillin G, and 100 mg/mL streptomycin (Invitrogen, Carlsbad, CA, USA). To identify factors that affect the proliferation and death of microglia, cell lines were cultured in their respective media, but with the addition of 0.2% FBS, 100 U/mL penicillin G, 100 mg/mL streptomycin (Invitrogen, Carlsbad, CA, USA), and either 10 ng/mL platelet-derived growth factor (PDGF)-AA, (PeproTech Inc., Rehovot, Israel), 10 ng/mL PDGF-BB (PeproTech), 10 ng/mL ciliary neurotrophic factor (CNTF; PeproTech Inc., Rehovot, Israel), 0.01–2.5 ng/mL TGF-β (PeproTech Inc., Rehovot, Israel), 10 ng/mL epidermal growth factor (EGF; PeproTech Inc., Rehovot, Israel), 10 ng/mL basic fibroblast growth factor (bFGF; PeproTech Inc., Rehovot, Israel), 0.1–30 ng/mL colony-stimulating factor 1 (CSF 1, M-CSF: R&D Systems, Minneapolis, MN, USA), or 5 mg/mL heparin [11].

### 2.2. Cell-Proliferation Assay

A 5-bromo-2′-deoxyuridine (BrdU)-based cell-proliferation enzyme-linked immunosorbent assay (ELISA) kit (Roche Diagnostics, Basel, Switzerland) was used to measure cell proliferation, according to the manufacturer’s protocol. Briefly, 48 h after the start of cultivation, BrdU labelling was performed. The BrdU-labelled DNA was stained with a peroxidase-conjugated anti-BrdU antibody and absorbance was measured at 450 nm using a microplate reader.

### 2.3. Quantitative Reverse-Transcription Polymerase Chain Reaction (qRT-PCR)

Total RNA from EOC 2 and SIM A9 cell lines was extracted using the High Pure RNA Isolation Kit (Roche, Basel, Switzerland) and then reverse-transcribed using Primer and enzyme mix (TOYOBO, Osaka, Japan). The cDNA samples were run minimally at three different concentrations in triplicate. All primers were designed using Primer3. qRT-PCR reactions were performed using SYBR Green on a MiniOpticon apparatus (both from BioRad, Hercules, CA, USA). The comparative Ct (ΔΔCt) method was used to determine the fold-change in expression using GAPDH (Glyceraldehyde 3-phosphate dehydrogenase) as the reference gene for normalization.

### 2.4. Cell Death-Detection Assay

The Cell Death Detection ELISA PLUS kit (Roche Diagnostics, Basel, Switzerland) was used to measure apoptotic cell death according to the manufacturer’s protocol. Due to TGF-β1 having the highest proliferative effect and bFGF having the lowest proliferative effect on microglia cells, we used these two growth factors to examine the apoptotic cell death on microglia. Briefly, TGF-β and bFGF were added to the respective wells individually or in combination, supplemented with 0.2% FBS. The plates were then incubated for 48 h at 37°C. Histone-complexed DNA fragments were stained with anti-histone and anti-DNA antibodies. Absorbance at 450 nm was measured using a microplate reader.

### 2.5. Cell Viability Analysis

Cells were seeded at a density of 1 × 10 ^3^ cells/well in 96-well plates and treated with 0.2% FBS, 10–100 µM galunisertib (MedChemExpress, Monmouth Junction, NJ, USA), or vehicle (10% FBS and 0.2% DMSO (Dimethyl sulfoxide), as a control) for 48, 72, or 96 h. Cell viability was evaluated using the WST-1 assay (2-(4-iodophenyl)-3-(4-nitrophenyl)-5-(2,4-disulfophenyl)-2H-tetrazolium, monosodium salt, Roche Diagnostics, Basel, Switzerland) for mitochondrial dehydrogenase activity, as previously described [12].

### 2.6. Statistical Analysis

Statistical analyses were carried out using the non-parametric Kruskal–Wallis test and the Steel–Dwass test was used for multiple comparisons. All statistical analyses were performed using Kyplot 5.0 (KyensLab Inc., Tokyo, Japan) and Microsoft Excel 2013 (Microsoft Corporation, Redmond, WA, USA). We considered *P* <0.05 to be statistically significant. Data are presented as the mean (standard deviation).

## 3. Results

### 3.1. Transforming Growth Factor Beta 1 Increases the Number of Microglial Cells In Vitro

**Figure 1:**
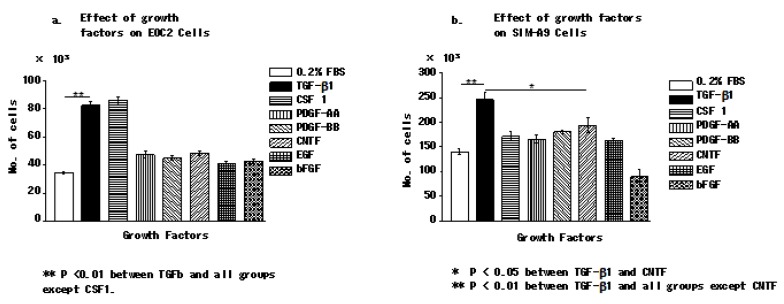
TGF-β promotes the proliferation of microglia.

To identify the signalling pathway that promotes microglial proliferation, we examined the effects of different growth factors on two mouse microglial cell lines, EOC 2 and SIM-A9. To eliminate the effect of FBS, 0.2% FBS was used for culturing. Among the growth factors; PDGF-AA, PDGF-BB, CNTF, TGF-β1, EGF, and bFGF; we found that only TGF-β1 (2.5 ng/mL) had a significant effect on cell proliferation (Figure 1a,b). Interestingly, FGF had a very weak proliferation-enhancing effect on the SIM A9 cell line, compared with control and other growth factors.

Treatment of the EOC 2 mouse microglial cell line with TGF-β and CSF 1 in 0.2% fetal bovine serum promoted the proliferation of EOC2 cells. TGF-β had a greater proliferation-enhancing effect on SIM A9 cells compared with the other growth factors. In contrast, bFGF had an inhibitory effect on the proliferation of SIM A9 cells. *P* < 0.05, was considered significant (Kruskal–Wallis or Steel–Dwass test). Data are presented as the mean of triplicate experiments; error bars represent the standard deviation.

### 3.2. Transforming Growth Factor Beta 1 and Colony-Stimulating Factor 1 Effectively Promote the Proliferation of Microglial Cells

**Figure 2:**
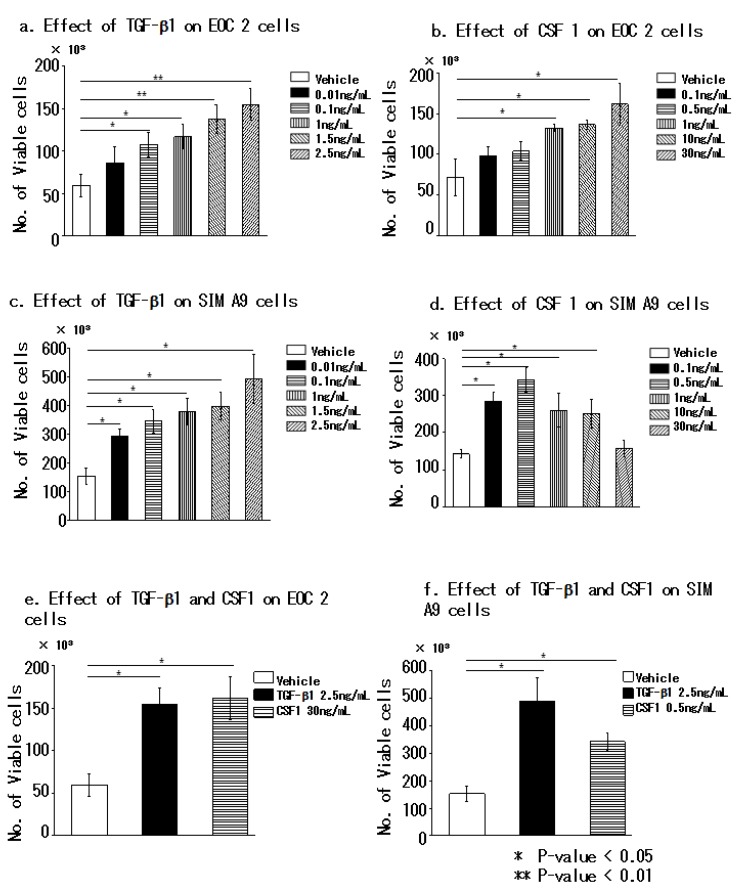
TGF-β and CSF 1 effectively promote the proliferation of microglia.

On the basis of recent studies demonstrating that spinal dorsal horn microgliosis is induced by CSF 1 [13,14], we compared the effects of TGF-β1 and CSF 1 on seeded EOC 2 (Figure 2a,b,e) and SIM A9 cells (Figure 2c,d,f). With either vehicle, 0.01–2.5 ng/mL TGF-β1 or 0.1–30 ng/mL CSF1 caused an increase in the proliferation of EOC 2 cells in a dose-dependent manner. With regard to SIM A9 cells, a dose-dependent increase in proliferation was observed in response to TGF-β1, but higher concentrations of CSF 1 had an inhibitory effect on these cells (Supplementary Figure S1). Comparison of the most effective proliferative doses revealed no statistically significant difference between the effects of TGF-β1 and CSF 1 (Figure 2e,f); thus, both factors have a proliferative effect on microglial cells.

Treatment of EOC 2 and SIM A9 mouse microglial cell lines with different concentrations of either TGF-β or CSF 1 revealed that both growth factors promoted the proliferation of EOC2 and SIM A9 cells. This experiment was performed in triplicate. *P* < 0.05, was considered significant (Kruskal–Wallis testor Steel–Dwass test). Data are presented as the mean of triplicate experiments; error bars represent the standard deviation.

### 3.3. Microglial Cell Lines Express Different Growth Factor Receptors

**Figure 3:**
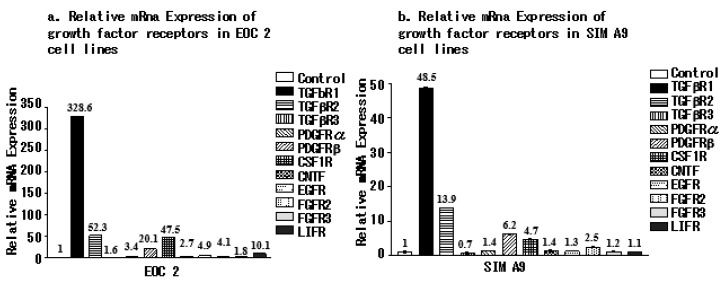
TGFβR2, TGFβR3, PDGFRα, PDGFR_β_, CSF1R, CNTFR, EGFR, FGFR2, FGFR3, and LIFR are expressed by both EOC 2 and SIM A9 cell lines. TGFβR1, TGFβR2, PDGFR_β_, and CSF1R were highly expressed in both cell lines compared with the other growth factors.

We next examined the expression of TGFβR1, TGFβR2, TGFβR3, PDGFRα, PDGFR_β_, CSF1R, CNTFR, EGFR, FGFR2, FGFR3, and LIFR in the microglial cell lines. qRT-PCR showed that all the growth factor receptors were expressed by the EOC 2 (Figure 3a) and SIM A9 cell lines (Figure 3b).

### 3.4. TGFβ-1 Inhibits Apoptotic Cell Death in EOC 2 Cells In Vitro

**Figure 4:**
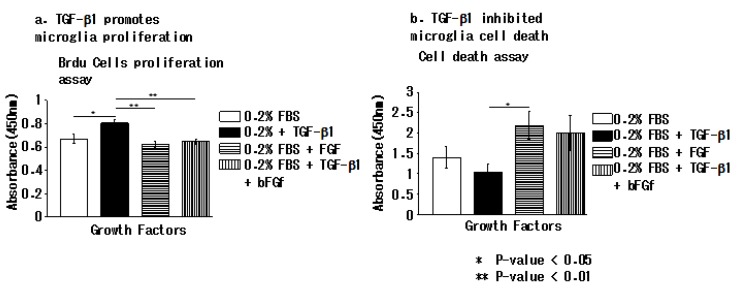
TGF-β promotes proliferation and inhibits cell death in microglia. (**a**) The bromodeoxyuridine cell proliferation assay revealed that TGF-β in 0.2% fetal bovine serum promoted the proliferation of microglial cells. (**b**) The cell-death assay showed that TGF-β in 0.2% fetal bovine serum inhibited microglial cell death. *P* < 0.05, was considered significant (Kruskal–Wallis test) Data are presented as the mean of triplicate experiments; error bars represent the standard deviation.

Next, we examined the in vitro proliferation and death of EOC 2 cells when cultured with 0.2% FBS only, TGF-β1, bFGF, or a combination of TGF-β1 and bFGF. The cell proliferation assay showed that TGF-β1 promoted EOC2 proliferation, while bFGF inhibited this TGF-β1-induced microglial proliferation (Figure 4a). The cell-death assay showed that only TGF-β1 inhibited cell death of EOC 2 cells cultured with 0.2% FBS (Figure 4b).

### 3.5. Inhibition of TGF-β1 by Galunisertib Reduces Microglial Proliferation

**Figure 5:**
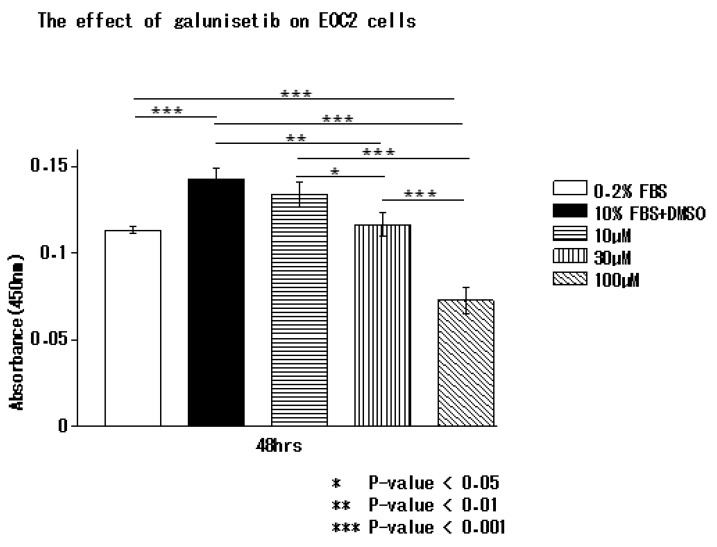
Galunisertib inhibits microglial proliferation in vitro. EOC 2 mouse microglial cells were treated with 0.2% FBS, 10% FBS + 0.2% DMSO, or 10, 30, or 100 µM galunisertib, a TGFβRI inhibitor. Galunisertib inhibited proliferation in a dose-dependent manner. The effect of galunisertib on EOC 2 cells was analysed by WST-1 assay at 0, 48, 72, and 96 h. *P* < 0.05, was considered significant (Kruskal–Wallis test). Data are presented as the mean of triplicate experiments; error bars represent the standard deviation.

Next, we assessed the effect of the TGFβRI inhibitor, galunisertib [10], on microglial proliferation in vitro. The WST-1 assay demonstrated that galunisertib inhibited the proliferation of EOC 2 cells in a dose-dependent manner (Figure 5).

## 4. Discussion

In the normal brain, TGF-β1 is present at very low concentrations, whereas its expression in activated glial cells is strongly increased in the injured brain. Microglia express and secrete TGF-β1 during injury, suggesting that these factors may mediate microglial activity in an autocrine or paracrine manner [15]. High levels of TGF-β1 have been implicated in microgliosis [16], astrogliosis [17], chronic hydrocephalus [18,19], vascular diseases [20], and fibrosis of the lung [21,22] and kidney [23].

In vitro, TGF-β1 has been shown to promote the survival of both neuronal and non-neural cells; Chalazonitis et al. showed that concentrations ranging from 0.1 to 100 ng/mL promote the proliferation of neurons [24]. Other studies have reported that CSF 1 promotes microglial proliferation [13]. In the present study, we demonstrate that TGF-β1 promotes the proliferation of microglial cells, suggesting that TGF-β1 has an effect similar to that of CSF 1 on microglial proliferation.

Inhibition of inappropriate hyperactivation of microglia after brain injury is important for neuroprotection and the recovery of damaged neurons. In line with our current results on the effects of galunisertib, a novel TGFβRI kinase inhibitor, SD-208, has been shown to alleviate brain injury and neurological deficits following germinal-matrix haemorrhage [25]. Administration of SB505124, an inhibitor of TGFβRI ALK5, to animals with neonatal hypoxic injury has been shown to inhibit both microglial and astrocytic proliferation, resulting in improved functional recovery [26]. Based on our current findings, we speculate that treatment with galunisertib may alleviate brain injury, central sensitization, and persistent pain following spinal cord injury. However, not all studies have reported a proliferative effect of TGF-β1 or an anti-proliferative effect of TGFβRI antagonism on glial cells [27]. Rozovsky et al. reported that TGF-β1 inhibited the proliferation of cultured astrocytes and microglia from 3-month-old, but not 24-month-old, rat brain [28]. In addition, both the absence and overproduction of TGF-β1 have been associated with microgliosis [16]. We speculate that the discrepancy may be attributable to differences in cell lines and experimental design and/or the concentration, timing, effectiveness, and selectivity of administered drugs. Further studies are needed to clarify the effects of TGF-β on glial cell proliferation in vitro and in vivo.

## 5. Conclusions

Our findings demonstrate that antagonism of TGF-β1 signalling inhibits the proliferation of microglia. The TGFβRI inhibitor galunisertib could, therefore, be a promising therapeutic agent for the treatment of neurodegenerative diseases associated with excessive microglial proliferation.

## Supplementary Materials

The following are available online at https://www.mdpi.com/2076-3425/10/1/20/s1, Figure S1: The effects of TGF-β1 and CSF on EOC 2 and SIM A9 cells.

## Abbreviations

bFGF	basic fibroblast growth factor
BrdU	5-bromo-2′-deoxyuridine
CSF 1	colony stimulating factor 1
CNS	central nervous system
CNTF	ciliary neurotrophic factor
DMEM	Dulbecco’s Modified Eagle Medium
EGF	epidermal growth factor
ELISA	enzyme-linked immunosorbent assay
FBS	fetal bovine serum
PDGF-AA	platelet-derived growth factor-AA
PDGF-BB	platelet-derived growth factor-BB
TGF-β	transforming growth factor β
TGFβRI	transforming growth factor β receptor type I
