# Supplementary material for: TGF-β Promotes the Proliferation of Microglia In Vitro
Source: Brain Sci. 2019 Dec 30;10(1):20. doi: 10.3390/brainsci10010020 (PMC7016844; doi:10.3390/brainsci10010020)

Supplementary figure 1.

a. Comparison on the effect of TGF-β1 1 and CSF 1 on EOC 2 cells

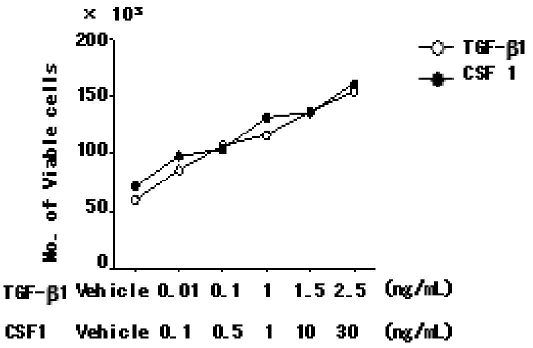

b. Comparison on the effect of TGF-β1 1 and CSF 1 on SIM A9 cells

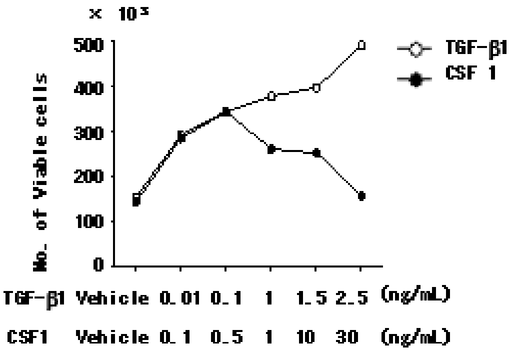

Supplement: Supplementary file 1 [file brainsci-10-00020-s001.pdf]
